# Relationship of living arrangement with the decline in functional capacity in elderly people by gender: a longitudinal observational study

**DOI:** 10.1186/s12199-020-00853-w

**Published:** 2020-05-20

**Authors:** Haruhiko Imamura, Eiko Uchiyama, Miki Akiyama, Ikuyo Kaneko, Toru Takebayashi, Yuji Nishiwaki

**Affiliations:** 1 Department of Environmental and Occupational Health, School of Medicine, Toho University, 5-21-16 Omori-Nishi, Ota-ku, Tokyo, 143-8540 Japan; 2 Graduate School of Media and Governance, Keio University, Kanagawa, Japan; 3 Faculty of Environment and Information Studies, Keio University, Kanagawa, Japan; 4 Keio University, Kanagawa, Japan; 5 Department of Preventive Medicine and Public Health, School of Medicine, Keio University, Tokyo, Japan

**Keywords:** Elderly, Functional capacity, Living arrangement, Longitudinal study, Spouse of a child

## Abstract

**Background:**

The living arrangement has been suggested as an important factor affecting health. Recent studies have also suggested that there was a risk among elderly persons who were not alone. This study examined whether the detailed living arrangement was associated with a future decline in functional capacity in the elderly, by gender, in a Japanese suburban city.

**Methods:**

A 3-year longitudinal questionnaire survey (baseline: 2011; follow-up: 2014) for aged 65 years or older was conducted in Kurihara city, Japan. Of the respondents in the baseline survey, we analyzed those who scored 13 points (a perfect score which indicates the highest functional capacity; *n* = 2627) on the Tokyo Metropolitan Institute of Gerontology Index of Competence at the baseline. The exposure was living arrangement at baseline, divided into five categories: “with spouse only,” “living alone,” “with child and his/her spouse,” “with child without his/her spouse,” and “with other family/person.” The outcome was the decline in functional capacity at the follow-up survey (score decreased to 10 points or less from 13 points).

**Results:**

Of the 2627 analyzed population, 1199 (45.6%) were men. The incidence of the decline was 5.8% in men and 5.9% in women. Multivariable logistic regression analyses adjusted for age, educational attainment, and health behavior and condition revealed that in women, the odds ratio of the decline was higher in living with child and his/her spouse (2.41, 95% confidence interval; 1.10–5.28) referring to living with spouse only. When adjusting activities inside and outside the home such as housework additionally, the association was attenuated to marginal significance (2.25, 0.98–5.18). No statistical significance was observed in men.

**Conclusions:**

These results suggested that living with child and spouse of a child was associated with the future decline in women’s functional capacity.

## Background

Family relationship and living arrangement are important social contexts affecting elderly health. Many previous studies have examined the effects of marital and cohabitation statuses, whether living alone or not, and the gender differences in these effects [1–9]. For example, elderly men who had a spouse or living with other family had lower levels of mortality, frailty, and depression compared with those who had no spouse or living alone; this case did not hold true for women [1, 2, 6–9].

Recently, several studies have also suggested that there is a risk among elderly persons who were not alone: persons who were living with other than spouse or partner had higher mortality, worse mental health, and lower physical function [10–16]. In addition, the gender difference in the association was noted. For example, even if married, living with other than the spouse or unmarried children was associated with worse mental health in women but not in men, suggesting the influence of specific living arrangements [17, 18]. If a specific living arrangement is more associated with family health as a social factor, it could contribute to the early detection of elderly persons who have risks of health decline. However, because of the limited number of studies that have examined detailed living arrangement with whom one lives, consistent results have not yet been revealed. Few studies have also focused on functional capacity as the outcome [12–16]. Higher-level functional capacity, as described at stages 5 to 7 Lawton’s hierarchical model [19], deteriorates before basic Activities of Daily Living (ADL); its maintenance is important for an independent life in the elderly [20].

This study aims to examine whether the living arrangement is associated with the future decline of functional capacity in elderly persons by a longitudinal survey conducted in a Japanese suburban city.

## Methods

### Study population

A 3-year longitudinal observational survey on health and daily life (baseline: 2011; follow-up: 2014), using a self-administered questionnaire with elderly people, was conducted in Kurihara city, Miyagi Prefecture, Japan. Kurihara city, which is in northeast Japan and has the largest area in Miyagi Prefecture, was established in 2005 by merging 10 municipalities (As of October 2010, 24,383 of its population of 76,851 were aged 65 years or older.). In this study, we extracted six of the 10 regions (former municipalities) as a study area with the cooperation of Kurihara City Hall. To obtain the representative sample of Kurihara city, these regions were extracted based on regional characteristics (classified as densely populated area, plains area, intermediary area between plains and mountains, or mountainous area based on population density and geographical conditions) and population size (classified as higher or lower than 7500 residents). The eligible population was all residents aged 65 years or older and lived in the extracted six regions at the time of each survey. Those who were facility residents and hospital inpatients were, however, excluded from the survey. At the baseline survey, we sent the questionnaire to 14,097 residents in February 2011 and received responses from 11,821 (a response rate of 83.9%). Of those, 8375 residents responded to a follow-up survey, which was conducted in January 2014. Both surveys were conducted using anonymized IDs, and the results of both surveys were linked. These processes were also conducted with the cooperation of Kurihara City Hall.

**Fig. 1:**
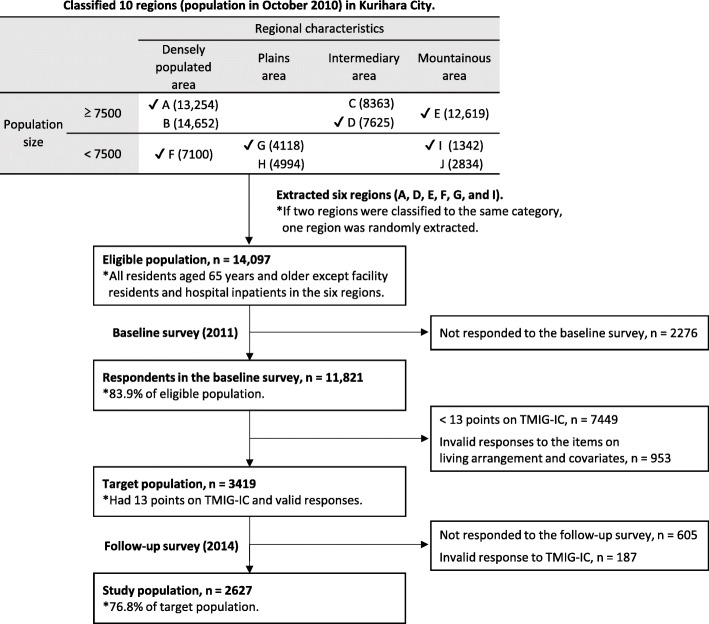
Conceptual framework for sampling the study population

Of the 11,821 individuals who responded to the baseline survey, 3419 were selected as the target population for this study, according to the following criteria: those who scored 13 points (perfect score) on the Tokyo Metropolitan Institute of Gerontology Index of Competence (TMIG-IC) and had valid responses to the items on living arrangement and covariates at the baseline. TMIG-IC measures higher-level competence in elderly people using 13 items. It comprises three subcategories, namely, instrumental self-maintenance (five items, e.g., “are you able to shop for daily necessities” or “are you able to prepare meals by yourself”), intellectual activity (four items, e.g., “are you able to fill out forms for your pension” or “do you read newspapers”), and social role (four items, e.g., “do you visit the homes of friends” or “are you sometimes called on for advice”) [20]. All questions are answerable by “yes” or “no.” Points are calculated by adding up the number of “yes” answers. The higher scores indicate higher functional capacity. In order to avoid reverse causality that low functional capacity had preceded a specific living arrangement at baseline, we selected well-performing respondents who had the highest scores. Of the 3419 respondents, 2814 (82.3%) responded to the follow-up survey; 2627 had a valid response to TMIG-IC at the follow-up as outcome measurement (final tracking rate of 76.8%). We analyzed these 2627 respondents in this study. Figure 1 illustrates the conceptual framework for the sampling process described above. The study protocol was approved by the Research Ethics Committee in Keio University Shonan Fujisawa Campus (No. 44) and the Ethics Committee of Faculty of Medicine, Toho University (No. 25104).

### Exposure measurement

The exposure in this study was the living arrangement at baseline. From the responses about persons with whom they were living, we classified the living arrangement into following five categories: “with spouse only,” “living alone,” “with child and his/her spouse,” “with child without his/her spouse,” “with other family/person.” These categories were set with reference to the reports of the national survey, Comprehensive Survey of Living Conditions, conducted by Japan’s Ministry of Health, Labour and Welfare [21]. With child and his/her spouse means that respondents were living with at least a child and his/her spouse. With child without his/her spouse refers to living with at least a child. These two categories included cases in which the spouse of respondent or other family is present. With other family/person involved the cases not applicable to the other four categories.

### Outcome measurement

The outcome measurement was the decline in functional capacity at the follow-up survey (about 3 years later). We defined “decline” as a decrease in TMIG-IC score to 10 points or less from 13 points at the baseline. This cutoff value was set based on the average value of Japanese people aged over 65 years [22] and used in the previous study [23].

### Covariates

In addition to age and sex, educational attainment, health behavior and condition (current drinking, current smoking, history of major diseases, and depressive symptoms), and activities inside and outside the home (housework, social participation, and relationship with neighbors) at the baseline survey were considered as covariates that might be related to the functional capacity of the elderly. Age was a continuous variable. The other covariates were dichotomized as follows: educational attainment (“≥10 years” and “<10 years”), current drinking (“no” and “yes”), current smoking (“no” and “yes”), history of major diseases (“no” and “yes”), depressive symptoms (“normal: 1 point or less out of 5 measured by the 5-item Geriatric Depression Scale [GDS5]” and “have depressive symptoms: 2 points or more on the GDS5”), housework (“mainly do” and “not mainly do”), social participation (“yes” and “no”), and relationship with neighbors (“frequent” and “not frequent”). History of major diseases was defined as having any one of the following diseases known to be causes of death or disability in older adults, referring to the previous study [24]: stroke, myocardial infarction/angina, diabetes, Parkinson’s disease, femoral neck fracture, and cancer. GDS5 was developed as a short form of the 30- or 15-item GDS and composed of 5 items such as life satisfaction and feeling of helplessness [25]. The validity of the GDS5 and the cutoff value (2 points or more) were verified [26]. In addition, GDS5 was showed to be associated with a future decline in ADL in the elderly [27]. Social participation was measured by the active members of a group/organization in the following four categories: local community groups; sports, hobby, or leisure group; voluntary organization or nonprofit organization; or other organizations. These categories were used in the previous study and associated with a future decline in ADL and death [28]. Though TMIG-IC includes a social role as a subcategory, we put activities inside and outside the home (housework, social participation, and relationship with neighbors) into covariates as indicators related to more independent activity at the home and social capital in the community [29].

### Statistical analysis

After stratification by gender, odds ratios (ORs) of the presence on outcome were estimated using a multivariable logistic regression analysis, to analyze whether the living arrangement at baseline was associated with the decline in functional capacity after 3 years. The reference category was “with spouse only,” based on previous studies [17, 30]. The reason for stratification by gender in the analyses is that there was a statistically significant interaction between living arrangement and sex on the outcome (*p* for interaction = 0.014 in the crude model). First, the association of each exposure variable with outcomes was assessed in the model adjusting for age (Model 1). Next, educational attainment, current drinking, current smoking, history of major diseases, and depressive symptoms were added to Model 1 (Model 2). Finally, activities inside and outside the home (housework, social participation, and relationship with neighbors) were included in the model (Model 3). In addition, the following three sensitivity analyses were conducted on Model 2. First, to examine whether ORs were changed when each covariate of activities inside and outside the home was added separately (sensitivity analysis 1). Second, to confirm whether the results did not depend on the cutoff value, analyses were conducted in which the cutoff value was changed from 10 points to 9 points and 11 points, respectively (sensitivity analysis 2). Third, the analyzed population was expanded from those who scored 13 points on the TMIG-IC to those who scored 11 points or more (sensitivity analysis 3). The statistical significance level was set at *p* < 0.05. All analyses were performed using STATA, version 14.0 (STATA Corporation, College Station, Texas).

## Results

Of the 2627 analyzed population, 1199 (45.6%) were men and 1428 (54.4%) were women. The mean age (±standard deviation) was 72.9 (±5.5) years for men and 72.6 (±5.1) years for women. Table 1 shows the baseline characteristics of the total study population and the population by gender. The proportion of five categories of living arrangement (i.e., “with spouse only,” “living alone,” “with child and his/her spouse,” “with child without his/her spouse,” and “with other family/person”) were 28.3%, 6.3%, 28.5%, 23.3%, and 13.6% in total. These proportions by gender were 30.9%, 3.4%, 29.1%, 23.3%, and 13.3%, respectively, for men, and 26.2%, 8.7%, 28.0%, 23.3%, and 13.8%, respectively, for women.

**Table 1:** Characteristics of the study population at baseline

Total	Living arrangement						
	With spouse only	Living alone	With child and his/her spouse	With child without his/her spouse		With other family/person	*P* value^a^
	***n*** = 744	***n*** = 165	***n*** = 749	***n*** = 612		***n*** = 357	
**Age (years)**							
65–69	266 (35.8%)	26 (15.8%)	197 (26.3%)	218 (35.6%)		129 (36.1%)	< 0.001
70–74	273 (36.7%)	57 (34.5%)	243 (32.4%)	207 (33.8%)		114 (31.9%)	
75–79	141 (19.0%)	49 (29.7%)	201 (26.8%)	116 (19.0%)		80 (22.4%)	
80–84	53 (7.1%)	26 (15.8%)	85 (11.3%)	52 (8.5%)		27 (7.6%)	
≥ 85	11 (1.5%)	7 (4.2%)	23 (3.1%)	19 (3.1%)		7 (2.0%)	
Mean ± standard deviation years	(72.0 ± 4.9)	(74.8 ± 5.3)	(73.5 ± 5.4)	(72.4 ± 5.4)		(72.2 ± 5.3)	
**Educational attainment (years)**							
≥ 10	493 (66.3%)	104 (63.0%)	416 (55.5%)	333 (54.4%)		184 (51.5%)	< 0.001
< 10	251 (33.7%)	61 (37.0%)	333 (44.5%)	279 (45.6%)		173 (48.5%)	
**Current drinking**							
No	433 (58.2%)	119 (72.1%)	461 (61.5%)	389 (63.6%)		217 (60.8%)	0.014
Yes	311 (41.8%)	46 (27.9%)	288 (38.5%)	223 (36.4%)		140 (39.2%)	
**Current smoking**							
No	692 (93.0%)	153 (92.7%)	699 (93.3%)	556 (90.8%)		326 (91.3%)	0.408
Yes	52 (7.0%)	12 (7.3%)	50 (6.7%)	56 (9.2%)		31 (8.7%)	
**History of major diseases**^b^							
No	588 (79.0%)	135 (81.8%)	590 (78.8%)	488 (79.7%)		292 (81.8%)	0.731
Yes	156 (21.0%)	30 (18.2%)	159 (21.2%)	124 (20.3%)		65 (18.2%)	
**Depressive symptoms (GDS5)**							
Normal (< 2 points)	656 (88.2%)	134 (81.2%)	666 (88.9%)	537 (87.7%)		310 (86.8%)	0.097
Have depressive symptoms (≥ 2 points)	88 (11.8%)	31 (18.8%)	83 (11.1%)	75 (12.3%)		47 (13.2%)	
**Housework**							
Mainly do	449 (60.3%)	156 (94.5%)	311 (41.5%)	356 (58.2%)		191 (53.5%)	< 0.001
Not mainly do	295 (39.7%)	9 (5.5%)	438 (58.5%)	256 (41.8%)		166 (46.5%)	
**Social participation**							
Yes	667 (89.7%)	127 (77.0%)	670 (89.5%)	545 (89.1%)		322 (90.2%)	< 0.001
No	77 (10.3%)	38 (23.0%)	79 (10.5%)	67 (10.9%)		35 (9.8%)	
**Relationship with neighbors**							
Frequent	710 (95.4%)	148 (89.7%)	735 (98.1%)	593 (96.9%)		345 (96.6%)	< 0.001
Not frequent	34 (4.6%)	17 (10.3%)	14 (1.9%)	19 (3.1%)		12 (3.4%)	
**Men**	***n*= 370**	***n*= 41**	***n*= 349**	***n*= 279**		***n*= 160**	
**Age (years)**							
65–69	106 (28.6%)	8 (19.5%)	82 (23.5%)	108 (38.7%)		66 (41.3%)	< 0.001
70–74	137 (37.0%)	14 (34.1%)	116 (33.2%)	90 (32.3%)		41 (25.6%)	
75–79	82 (22.2%)	10 (24.4%)	95 (27.2%)	49 (17.6%)		37 (23.1%)	
80–84	35 (9.5%)	5 (12.2%)	42 (12.0%)	27 (9.7%)		12 (7.5%)	
≥ 85	10 (2.7%)	4 (9.8%)	14 (4.0%)	5 (1.8%)		4 (2.5%)	
Mean ± standard deviation years	(72.9 ± 5.2)	(74.7 ± 6.2)	(73.9 ± 5.6)	(72.1 ± 5.3)		(72.0 ± 5.5)	
**Educational attainment (years)**							
≥ 10	246 (66.5%)	23 (56.1%)	187 (53.6%)	155 (55.6%)		80 (50.0%)	0.001
< 10	124 (33.5%)	18 (43.9%)	162 (46.4%)	124 (44.4%)		80 (50.0%)	
**Current drinking**							
No	117 (31.6%)	14 (34.1%)	118 (33.8%)	93 (33.3%)		49 (30.6%)	0.940
Yes	253 (68.4%)	27 (65.9%)	231 (66.2%)	186 (66.7%)		111 (69.4%)	
**Current smoking**							
No	321 (86.8%)	31 (75.6%)	301 (86.2%)	226 (81.0%)		133 (83.1%)	0.111
Yes	49 (13.2%)	10 (24.4%)	48 (13.8%)	53 (19.0%)		27 (16.9%)	
**History of major diseases**^b^							
No	269 (72.7%)	31 (75.6%)	256 (73.4%)	201 (72.0%)		117 (73.1%)	0.990
Yes	101 (27.3%)	10 (24.4%)	93 (26.6%)	78 (28.0%)		43 (26.9%)	
**Depressive symptoms (GDS5)**							
Normal (< 2 points)	332 (89.7%)	30 (73.2%)	308 (88.3%)	253 (90.7%)		136 (85.0%)	0.011
Have depressive symptoms (≥ 2 points)	38 (10.3%)	11 (26.8%)	41 (11.7%)	26 (9.3%)		24 (15.0%)	
**Housework**							
Mainly do	84 (22.7%)	35 (85.4%)	59 (16.9%)	69 (24.7%)		42 (26.3%)	< 0.001
Not mainly do	286 (77.3%)	6 (14.6%)	290 (83.1%)	210 (75.3%)		118 (73.8%)	
**Social participation**							
Yes	340 (91.9%)	36 (87.8%)	328 (94.0%)	255 (91.4%)		150 (93.8%)	0.485
No	30 (8.1%)	5 (12.2%)	21 (6.0%)	24 (8.6%)		10 (6.3%)	
**Relationship with neighbors**							
Frequent	351 (94.9%)	36 (87.8%)	342 (98.0%)	265 (95.0%)		151 (94.4%)	0.020
Not frequent	19 (5.1%)	5 (12.2%)	7 (2.0%)	14 (5.0%)		9 (5.6%)	
**Women**	***n*= 374**	***n*= 124**	***n*= 400**	***n*= 333**		***n*= 197**	
**Age (years)**							
65–69	160 (42.8%)	18 (14.5%)	115 (28.8%)	110 (33.0%)		63 (32.0%)	< 0.001
70–74	136 (36.4%)	43 (34.7%)	127 (31.8%)	117 (35.1%)		73 (37.1%)	
75–79	59 (15.8%)	39 (31.5%)	106 (26.5%)	67 (20.1%)		43 (21.8%)	
80–84	18 (4.8%)	21 (16.9%)	43 (10.8%)	25 (7.5%)		15 (7.6%)	
≥ 85	1 (0.3%)	3 (2.4%)	9 (2.3%)	14 (4.2%)		3 (1.5%)	
Mean ± standard deviation years	(71.0 ± 4.2)	(74.9 ± 5.1)	(73.2 ± 5.2)	(72.8 ± 5.5)		(72.4 ± 5.1)	
**Educational attainment (years)**							
≥ 10	247 (66.0%)	81 (65.3%)	229 (57.3%)	178 (53.5%)		104 (52.8%)	0.002
< 10	127 (34.0%)	43 (34.7%)	171 (42.8%)	155 (46.5%)		93 (47.2%)	
**Current drinking**							
No	316 (84.5%)	105 (84.7%)	343 (85.8%)	296 (88.9%)		168 (85.3%)	0.509
Yes	58 (15.5%)	19 (15.3%)	57 (14.3%)	37 (11.1%)		29 (14.7%)	
**Current smoking**							
No	371 (99.2%)	122 (98.4%)	398 (99.5%)	330 (99.1%)		193 (98.0%)	0.428
Yes	3 (0.8%)	2 (1.6%)	2 (0.5%)	3	(0.9%)	4 (2.0%)	
**History of major diseases**^b^							
No	319 (85.3%)	104 (83.9%)	334 (83.5%)	287 (86.2%)		175 (88.8%)	0.491
Yes	55 (14.7%)	20 (16.1%)	66 (16.5%)	46 (13.8%)		22 (11.2%)	
**Depressive symptoms (GDS5)**							
Normal (< 2 points)	324 (86.6%)	104 (83.9%)	358 (89.5%)	284 (85.3%)		174 (88.3%)	0.340
Have depressive symptoms (≥ 2 points)	50 (13.4%)	20 (16.1%)	42 (10.5%)	49 (14.7%)		23 (11.7%)	
**Housework**							
Mainly do	365 (97.6%)	121 (97.6%)	252 (63.0%)	287 (86.2%)		149 (75.6%)	< 0.001
Not mainly do	9 (2.4%)	3 (2.4%)	148 (37.0%)	46 (13.8%)		48 (24.4%)	
**Social participation**							
Yes	327 (87.4%)	91 (73.4%)	342 (85.5%)	290 (87.1%)		172 (87.3%)	0.002
No	47 (12.6%)	33 (26.6%)	58 (14.5%)	43 (12.9%)		25 (12.7%)	
**Relationship with neighbors**							
Frequent	359 (96.0%)	112 (90.3%)	393 (98.3%)	328 (98.5%)		194 (98.5%)	< 0.001
Not frequent	15 (4.0%)	12 (9.7%)	7 (1.8%)	5 (1.5%)		3 (1.5%)	

^a^Chi-square test

^b^History of major diseases was defined as having any one of the following diseases: stroke, myocardial infarction/angina, diabetes, Parkinson’s disease, femoral neck fracture, and cancer

Table 2 shows the association between the living arrangement at the baseline and functional capacity at the follow-up for men. Among those analyzed, 5.8% (69/1199) had the decline in functional capacity after 3 years. The incidence of the decline was lowest in “with child without his/her spouse” (4.7%) and highest in “living alone” (9.8%). However, no clear association was observed in all models.

**Table 2:** Association between living arrangement and decline in functional capacity after three years for men (*n* = 1199)

	Outcome/study population (%)	Crude	Model 1^b^	Model 2^c^	Model 3^d^
		OR (95% CI)^a^	OR (95% CI)	OR (95% CI)	OR (95% CI)
**Living arrangement**					
With spouse only	19/370 (5.1%)	1	1	1	1
Living alone	4/41 (9.8%)	2.00 (0.65–6.18)	1.56 (0.49–5.00)	1.30 (0.40–4.27)	1.85 (0.51–6.72)
With child and his/her spouse	20/349 (5.7%)	1.12 (0.59–2.14)	0.97 (0.50–1.88)	0.85 (0.44–1.68)	0.89 (0.45–1.76)
With child without his/her spouse	13/279 (4.7%)	0.90 (0.44–1.86)	0.99 (0.47–2.06)	0.88 (0.42–1.86)	0.93 (0.44–1.98)
With other family/person	13/160 (8.1%)	1.63 (0.79–3.39)	1.84 (0.87–3.88)	1.61 (0.75–3.47)	1.65 (0.76–3.58)
**Age**					
Continuous	-		1.13 (1.09–1.18)	1.11 (1.06–1.16)	1.11 (1.06–1.17)
**Educational attainment (years)**					
≥ 10	25/691 (3.6%)			1	1
< 10	44/508 (8.7%)			1.83 (1.05–3.17)	1.85 (1.06–3.23)
**Current drinking**					
No	37/391 (9.5%)			1	1
Yes	32/808 (4.0%)			0.48 (0.29–0.80)	0.49 (0.29–0.82)
**Current smoking**					
No	58/1012 (5.7%)			1	1
Yes	11/187 (5.9%)			1.52 (0.76–3.06)	1.37 (0.67–2.78)
**History of major diseases**					
No	48/874 (5.5%)			1	1
Yes	21/325 (6.5%)			1.12 (0.64–1.97)	1.06 (0.60–1.88)
**Depressive symptoms (GDS5)**					
Normal (< 2 points)	53/1059 (5.0%)			1	1
Have depressive symptoms (≥ 2 points)	16/140 (11.4%)			2.40 (1.28–4.50)	2.08 (1.09–3.98)
**Housework**					
Mainly do	13/289 (4.5%)				1
Not mainly do	56/910 (6.2%)				1.74 (0.85–3.56)
**Social participation**					
Yes	57/1109 (5.1%)				1
No	12/90 (13.3%)				1.27 (0.60–2.70)
**Relationship with neighbors**					
Frequent	60/1145 (5.2%)				1
Not frequent	9/54 (16.7%)				3.27 (1.41–7.61)

*OR* odds ratio, *CI* confidence interval

^a^Adjusted OR and 95% CI were estimated by multivariate logistic regression

^b^Model 1: adjusted for age (continuous)

^c^Model 2: adjusted for age (continuous), educational attainment, current drinking, current smoking, history of major diseases, and depressive symptoms

^d^Model 3: adjusted for age (continuous), educational attainment, current drinking, current smoking, history of major diseases, depressive symptoms, housework, social participation, and relationship with neighbors

Table 3 shows the association between the living arrangement at the baseline and functional capacity at the follow-up for women. Among those analyzed, 5.9% (84/1428) had the decline in functional capacity after 3 years. The incidence of the decline was lowest in “with spouse only” (2.4%) and highest in “with child and his/her spouse” (8.8%). Multivariable logistic regression analyses showed that the adjusted OR was 2.41 (95% confidence interval; 1.10–5.28) in “with child and his/her spouse” in Model 2. Including activities inside and outside the home slightly attenuated the association (Model 3, 2.25, 0.98–5.18), but still remained a marginal significance. There were also high ORs in “with other family/person,” but not with statistical significance.

**Table 3:** Association between living arrangement and decline in functional capacity after three years for women (*n* = 1428)

	Outcome/study population (%)	Crude	Model1^b^	Model2^c^	Model3^d^
		OR (95% CI)^a^	OR (95% CI)	OR (95% CI)	OR (95% CI)
**Living arrangement**					
With spouse only	9/374 (2.4%)	1	1	1	1
Living alone	9/124 (7.3%)	3.17 (1.23–8.19)	1.49 (0.56–3.99)	1.68 (0.62–4.56)	1.64 (0.60–4.47)
With child and his/her spouse	35/400 (8.8%)	3.89 (1.84–8.21)	2.47 (1.14–5.33)	2.41 (1.10–5.28)	2.25 (0.98–5.18)
With child without his/her spouse	16/333 (4.8%)	2.05 (0.89–4.70)	1.20 (0.51–2.87)	1.02 (0.42–2.50)	1.05 (0.42–2.60)
With other family/person	15/197 (7.6%)	3.34 (1.44–7.78)	2.41 (1.00–5.79)	2.36 (0.96–5.83)	2.27 (0.89–5.78)
**Age**					
Continuous	-		1.21 (1.16–1.26)	1.21 (1.16–1.27)	1.20 (1.15–1.26)
**Educational attainment (years)**					
≥ 10	30/839 (3.6%)			1	1
< 10	54/589 (9.2%)			2.19 (1.33–3.61)	2.21 (1.34–3.66)
**Current drinking**					
No	76/1228 (6.2%)			1	1
Yes	8/200 (4.0%)			0.89 (0.41–1.96)	0.90 (0.41–2.00)
**Current smoking**					
No	83/1414 (5.9%)			1	1
Yes	1/14 (7.1%)			1.20 (0.13–11.25)	1.40 (0.15–13.06)
**History of major diseases**					
No	60/1219 (4.9%)			1	1
Yes	24/209 (11.5%)			2.70 (1.56–4.67)	2.78 (1.60–4.83)
**Depressive symptoms (GDS5)**					
Normal (< 2 points)	61/1244 (4.9%)			1	1
Have depressive symptoms (≥ 2 points)	23/184 (12.5%)			2.81 (1.57–5.01)	2.61 (1.44–4.72)
**Housework**					
Mainly do	50/1174 (4.3%)				1
Not mainly do	34/254 (13.4%)				1.32 (0.75–2.34)
**Social participation**					
Yes	66/1222 (5.4%)				1
No	18/206 (8.7%)				1.10 (0.59–2.04)
**Relationship with neighbors**					
Frequent	79/1386 (5.7%)				1
Not frequent	5/42 (11.9%)				3.37 (1.14–9.96)

*OR* odds ratio, *CI* confidence interval

^a^Adjusted OR and 95% CI were estimated by multivariate logistic regression

^b^Model 1: adjusted for age (continuous)

^c^Model 2: adjusted for age (continuous), educational attainment, current drinking, current smoking, history of major diseases, and depressive symptoms

^d^Model 3: adjusted for age (continuous), educational attainment, current drinking, current smoking, history of major diseases, depressive symptoms, housework, social participation, and relationship with neighbors

**Fig. 2:**
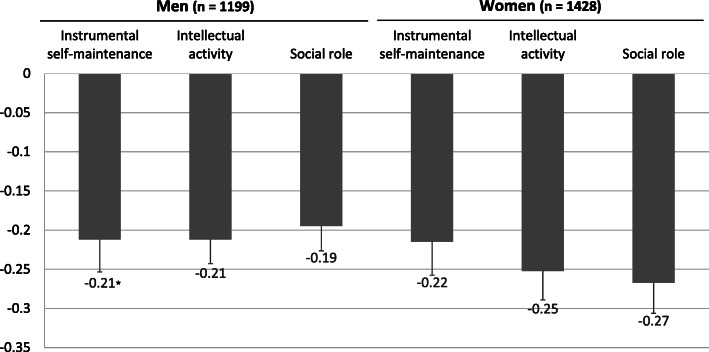
Mean decrease score of the three subcategories of the Tokyo Metropolitan Institute of Gerontology Index of Competence (TMIG-IC) in those living with child and his/her spouse. *Mean and standard error

**Fig. 3:**
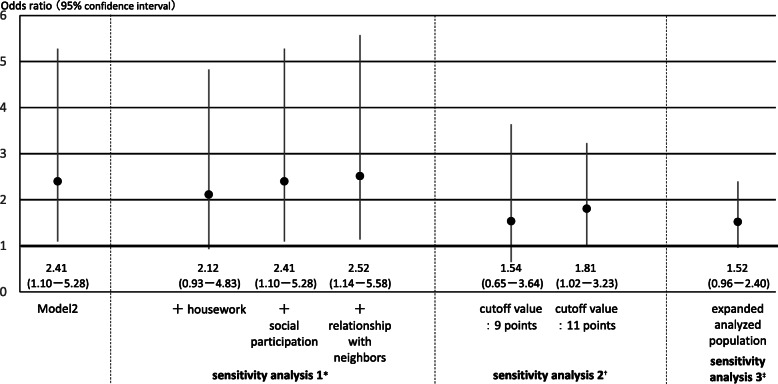
Three sensitivity analyses based on Model 2 in those living with child and his/her spouse among women. *Results when each covariate of activities inside and outside the home was added separately. ^†^Results when the cutoff value was changed from 10 points on the TMIG-IC to 9 points and 11 points, respectively. ^‡^Result when the analyzed population was expanded from those who scored 13 points on the TMIG-IC to those who scored 11 points or more (*n* = 2173) with adding baseline scores to covariates

To examine which subcategory of TMIG-IC was affected in “living with child and his/her spouse,” the mean decrease value of the three subcategories at the follow-up survey was calculated (Fig. 2). In women, although the decrease was shown in all subcategories, social role decreased most (− 0.27 out of 4 points) compared with instrumental self-maintenance (− 0.22 out of 5 points) and intellectual activity (− 0.25 out of 4 points). In addition, three sensitivity analyses were conducted for those living “with child and his/her spouse” among women on Model 2 (Fig. 3). First, after adding each covariate of activities inside and outside the home to Model 2 separately, the OR was slightly reduced when adding housework (sensitivity analysis 1). Second, the ORs were reduced but still remained a significance (1.81, 1.02–3.23) when the cutoff value was changed to 11 points on the TMIG-IC (sensitivity analysis 2). Third, the OR was also slightly reduced but still remained a marginal significance (1.52, 0.96–2.40) when expanding the analyzed population to those who scored 11 points or more on the TMIG-IC (*n* = 2173 in women) and adding baseline TMIG-IC scores to the covariates (sensitivity analysis 3).

## Discussion

The results of multivariable logistic regression analyses adjusting all covariates revealed that, in women, the decline in functional capacity is most associated with living with a child and his/her spouse, the category with the highest proportion. No statistically significant association was found in men.

Previous studies showed that men who were not married or living alone had higher mortality [1, 2, 6, 8, 10, 11], worse mental health [9, 17, 30], functional disability [12], and frailty [7], suggesting the protective effects of spouse and other families on health. For elderly men, the spouse could play the role of caretakers, providers of health-related information, and inhibitors of unhealthy behaviors [6], while living alone was associated with a lack of social support and social isolation, which could lead to emotional stress and depression, and subsequent decline in ADL [9, 27, 31]. In this study, in men, though there was no statistically significant association between living arrangement and the decline in functional capacity, OR of “living alone” was the highest (1.85 in Model 3). As the number of persons living alone was quite small in men (*n* = 41), it is difficult to determine whether the result was caused by the small sample number or indicated no association truly. In fact, however, men who lived alone were more likely to have depressive symptoms and not to have a frequent relationship with neighbors (Table 1). Further studies with long follow-up time in large-scale would make the result clearer. On the other hand, in women, although the OR of “living alone” was higher than that of “with spouse only,” the highest OR was shown in “with child and his/her spouse,” regardless of the presence or absence of a spouse. Studies have indicated that living with other than a spouse was associated with worse mental health [16–18] and mobility limitation [14] in women. These results suggested that a specific relationship with other families might be a risk for women. In this study, considering that there was no association in “with child without his/her spouse,” an important factor could be the spouse of the child.

One hypothesis that explains the mechanism behind the negative effect of a certain living arrangement on functional capacity in women is the excessive support in the household. In Japan, the traditional norm is that elderly people depend on informal care, which is provided by younger generations, specifically by daughters-in-law (spouse of child) [32, 33]. Elderly women who were living with child and his/her spouse might not necessarily have to shop or go out by themselves, because their child and his/her spouse act on their behalf, resulting in inhibiting the independent daily living. In that case, the influence might appear on a specific aspect, such as instrumental ADL. However, when focusing on the three subcategories of TMIG-IC (instrumental self-maintenance, intellectual activity, and social role), the decreased score was shown in all three subcategories (Fig. 2). From the results of sensitivity analyses (Fig. 3), the high OR in “with child and his/her spouse” was partly explained by the housework. It might be important to have an active role at home for elderly women in this situation to prevent a decline in functional capacity.

Another hypothesis is that the result might be caused by the isolation of women in and outside the household. A recent study showed that elderly women who were not living alone but eating alone in daily life had a higher risk of depression; this was not a case in men [34]. The relationship with the spouse of a child could be a burden at times, resulting in the isolation of elderly women. Regarding nursing care, studies have showed the difference of effect on elderly health by the relationship with a caregiver [33, 35]. For example, women who received care by daughter-in-law (spouse of a child) had higher mortality than those who received care by spouse [33]. Because this study targeted elderly persons with good functional capacity and there was no information on the gender of persons living together, a careful interpretation and follow-up study are necessary. It may also be useful if there was information on the means of transportation which respondents can use to go out by themselves.

The policy implication of the results is that health welfare policymakers should also focus and develop countermeasures to health risks in the elderly who are not living alone. For example, it may be necessary to strengthen home visits by a public health nurse for the early detection of elderly persons who have risks. In addition, this study suggests that the risk for the household as a whole, because if the elderly could not maintain an independent life and have a care need by their family, caregiving may pose risk to caregiver health [36].

Among the major strengths of this study are the relatively high response rate of the baseline survey (83.9%) and the tracking rate of the analyzed population (76.8%), which could reflect the actual condition of the elderly people in the study area well. Moreover, to the best of our knowledge, our study was the first that examined the association between detailed the living arrangement and higher-level functional capacity by gender using a longitudinal survey. This study has limitations, however. First, in this study, due to the low incidence of outcomes, point estimates were based on wide confidence intervals. This might mean that the follow-up time was too short or that the sample size was not large, as mentioned in the results for men, enough to catch the outcome in the statistical stability. In addition, the results of this study are partially unstable due to the cutoff value (Fig. 3). Further studies with long follow-up time in large-scale are needed in the future. Second, there was no information on the relationship of each household member. For example, women might have a good relationship with a child or his/her spouse, or not. However, when considering the relationship, the strength of the association, which was shown in this study, would be likely clearer. Third, we could not evaluate changes in the living arrangement during the follow-up period. Forth, due to data collection constraints, no information was available on loss to follow-up. Further studies are necessary in this regard. Finally, the generalizability of the results of this study might be an issue, as it was conducted in one city. However, Kurihara city has the largest and diversified area in Miyagi Prefecture, such as a densely populated area where a bullet train station is located or a mountainous area. Despite these limitations, this study provided evidence implying that the functional capacity of elderly persons can be affected by the living arrangement with whom one lives.

## Conclusions

In conclusion, living with child and his/her spouse was associated with the future decline in functional capacity for women, whereas the association was not statistically significant for men. The results of this study suggested that specific living arrangement other than living alone was a potential risk for higher functional capacity in the elderly and that the association varied by gender.

## Data Availability

The datasets generated and analyzed during the current study are not publicly available due to the contract with Kurihara City Hall regarding information protection.

## Abbreviations

**ADL**: Activities of Daily Living
**CI**: Confidence interval
**GDS5**: 5-Item Geriatric Depression Scale
**OR**: Odds ratio
**TMIG-IC**: Tokyo Metropolitan Institute of Gerontology Index of Competence
