# Correction to: Relationship of living arrangement with the decline in functional capacity in elderly people by gender: a longitudinal observational study

**DOI:** 10.1186/s12199-020-00860-x

**Published:** 2020-06-19

**Authors:** Haruhiko Imamura, Eiko Uchiyama, Miki Akiyama, Ikuyo Kaneko, Toru Takebayashi, Yuji Nishiwaki

**Affiliations:** 1 Department of Environmental and Occupational Health, School of Medicine, Toho University, 5–21-16 Omori-Nishi, Ota-ku, Tokyo, 143–8540 Japan; 2 Graduate School of Media and Governance, Keio University, Kanagawa, Japan; 3 Faculty of Environment and Information Studies, Keio University, Kanagawa, Japan; 4 Keio University, Kanagawa, Japan; 5 Department of Preventive Medicine and Public Health, School of Medicine, Keio University, Tokyo, Japan

**Correction to: Environ Health Prev Med (2020) 25: 15**

**https://doi.org/10.1186/s12199-020-00853-w**

Following publication of the original article [1], Table 1 was incorrectly published. Table 1 consists of three parts: Total, Men and Women; however, the first part “Total (n = 744, n = 165, n = 749, n = 612, n = 357)” was captured incorrectly as part of the header. It should be moved to the top row of the table just like the Men and Women. The correct Table 1 is shown below.

**Table 1:** Characteristics of the study population at baseline

	Living arrangement										
	With spouse only		Living alone		With child and his/her spouse		With child without his/her spouse		With other family/person		*P*-value ^a^
**Total**	***n*** **= 744**		***n*** **= 165**		***n*** **= 749**		***n*** **= 612**		***n*** **= 357**		
**Age (years)**											
65–69	266	(35.8%)	26	(15.8%)	197	(26.3%)	218	(35.6%)	129	(36.1%)	< 0.001
70–74	273	(36.7%)	57	(34.5%)	243	(32.4%)	207	(33.8%)	114	(31.9%)	
75–79	141	(19.0%)	49	(29.7%)	201	(26.8%)	116	(19.0%)	80	(22.4%)	
80–84	53	(7.1%)	26	(15.8%)	85	(11.3%)	52	(8.5%)	27	(7.6%)	
≥ 85	11	(1.5%)	7	(4.2%)	23	(3.1%)	19	(3.1%)	7	(2.0%)	
(Mean ± standard deviation years)	(72.0	±4.9)	(74.8	±5.3)	(73.5	±5.4)	(72.4	±5.4)	(72.2	±5.3)	
**Educational attainment (years)**											
≥ 10	493	(66.3%)	104	(63.0%)	416	(55.5%)	333	(54.4%)	184	(51.5%)	< 0.001
< 10	251	(33.7%)	61	(37.0%)	333	(44.5%)	279	(45.6%)	173	(48.5%)	
**Current drinking**											
No	433	(58.2%)	119	(72.1%)	461	(61.5%)	389	(63.6%)	217	(60.8%)	0.014
Yes	311	(41.8%)	46	(27.9%)	288	(38.5%)	223	(36.4%)	140	(39.2%)	
**Current smoking**											
No	692	(93.0%)	153	(92.7%)	699	(93.3%)	556	(90.8%)	326	(91.3%)	0.408
Yes	52	(7.0%)	12	(7.3%)	50	(6.7%)	56	(9.2%)	31	(8.7%)	
**History of major diseases**^**b**^											
No	588	(79.0%)	135	(81.8%)	590	(78.8%)	488	(79.7%)	292	(81.8%)	0.731
Yes	156	(21.0%)	30	(18.2%)	159	(21.2%)	124	(20.3%)	65	(18.2%)	
**Depressive symptoms (GDS5)**											
Normal (< 2 points)	656	(88.2%)	134	(81.2%)	666	(88.9%)	537	(87.7%)	310	(86.8%)	0.097
Have depressive symptoms (≥2 points)	88	(11.8%)	31	(18.8%)	83	(11.1%)	75	(12.3%)	47	(13.2%)	
**Housework**											
Mainly do	449	(60.3%)	156	(94.5%)	311	(41.5%)	356	(58.2%)	191	(53.5%)	< 0.001
Not mainly do	295	(39.7%)	9	(5.5%)	438	(58.5%)	256	(41.8%)	166	(46.5%)	
**Social participation**											
Yes	667	(89.7%)	127	(77.0%)	670	(89.5%)	545	(89.1%)	322	(90.2%)	< 0.001
No	77	(10.3%)	38	(23.0%)	79	(10.5%)	67	(10.9%)	35	(9.8%)	
**Relationship with neighbors**											
Frequent	710	(95.4%)	148	(89.7%)	735	(98.1%)	593	(96.9%)	345	(96.6%)	< 0.001
Not frequent	34	(4.6%)	17	(10.3%)	14	(1.9%)	19	(3.1%)	12	(3.4%)	
**Men**	***n*** **= 370**		***n*** **= 41**		***n*** **= 349**		***n*** **= 279**		***n*** **= 160**		
**Age (years)**											
65–69	106	(28.6%)	8	(19.5%)	82	(23.5%)	108	(38.7%)	66	(41.3%)	< 0.001
70–74	137	(37.0%)	14	(34.1%)	116	(33.2%)	90	(32.3%)	41	(25.6%)	
75–79	82	(22.2%)	10	(24.4%)	95	(27.2%)	49	(17.6%)	37	(23.1%)	
80–84	35	(9.5%)	5	(12.2%)	42	(12.0%)	27	(9.7%)	12	(7.5%)	
≥ 85	10	(2.7%)	4	(9.8%)	14	(4.0%)	5	(1.8%)	4	(2.5%)	
(Mean ± standard deviation years)	(72.9	±5.2)	(74.7	±6.2)	(73.9	±5.6)	(72.1	±5.3)	(72.0	±5.5)	
**Educational attainment (years)**											
≥ 10	246	(66.5%)	23	(56.1%)	187	(53.6%)	155	(55.6%)	80	(50.0%)	0.001
< 10	124	(33.5%)	18	(43.9%)	162	(46.4%)	124	(44.4%)	80	(50.0%)	
**Current drinking**											
No	117	(31.6%)	14	(34.1%)	118	(33.8%)	93	(33.3%)	49	(30.6%)	0.940
Yes	253	(68.4%)	27	(65.9%)	231	(66.2%)	186	(66.7%)	111	(69.4%)	
**Current smoking**											
No	321	(86.8%)	31	(75.6%)	301	(86.2%)	226	(81.0%)	133	(83.1%)	0.111
Yes	49	(13.2%)	10	(24.4%)	48	(13.8%)	53	(19.0%)	27	(16.9%)	
**History of major diseases**^**b**^											
No	269	(72.7%)	31	(75.6%)	256	(73.4%)	201	(72.0%)	117	(73.1%)	0.990
Yes	101	(27.3%)	10	(24.4%)	93	(26.6%)	78	(28.0%)	43	(26.9%)	
**Depressive symptoms (GDS5)**											
Normal (< 2 points)	332	(89.7%)	30	(73.2%)	308	(88.3%)	253	(90.7%)	136	(85.0%)	0.011
Have depressive symptoms (≥2 points)	38	(10.3%)	11	(26.8%)	41	(11.7%)	26	(9.3%)	24	(15.0%)	
**Housework**											
Mainly do	84	(22.7%)	35	(85.4%)	59	(16.9%)	69	(24.7%)	42	(26.3%)	< 0.001
Not mainly do	286	(77.3%)	6	(14.6%)	290	(83.1%)	210	(75.3%)	118	(73.8%)	
**Social participation**											
Yes	340	(91.9%)	36	(87.8%)	328	(94.0%)	255	(91.4%)	150	(93.8%)	0.485
No	30	(8.1%)	5	(12.2%)	21	(6.0%)	24	(8.6%)	10	(6.3%)	
**Relationship with neighbors**											
Frequent	351	(94.9%)	36	(87.8%)	342	(98.0%)	265	(95.0%)	151	(94.4%)	0.020
Not frequent	19	(5.1%)	5	(12.2%)	7	(2.0%)	14	(5.0%)	9	(5.6%)	
**Women**	***n*** **= 374**		***n*** **= 124**		***n*** **= 400**		***n*** **= 333**		***n*** **= 197**		
**Age (years)**											
65–69	160	(42.8%)	18	(14.5%)	115	(28.8%)	110	(33.0%)	63	(32.0%)	< 0.001
70–74	136	(36.4%)	43	(34.7%)	127	(31.8%)	117	(35.1%)	73	(37.1%)	
75–79	59	(15.8%)	39	(31.5%)	106	(26.5%)	67	(20.1%)	43	(21.8%)	
80–84	18	(4.8%)	21	(16.9%)	43	(10.8%)	25	(7.5%)	15	(7.6%)	
≥ 85	1	(0.3%)	3	(2.4%)	9	(2.3%)	14	(4.2%)	3	(1.5%)	
(Mean ± standard deviation years)	(71.0	±4.2)	(74.9	±5.1)	(73.2	±5.2)	(72.8	±5.5)	(72.4	±5.1)	
**Educational attainment (years)**											
≥ 10	247	(66.0%)	81	(65.3%)	229	(57.3%)	178	(53.5%)	104	(52.8%)	0.002
< 10	127	(34.0%)	43	(34.7%)	171	(42.8%)	155	(46.5%)	93	(47.2%)	
**Current drinking**											
No	316	(84.5%)	105	(84.7%)	343	(85.8%)	296	(88.9%)	168	(85.3%)	0.509
Yes	58	(15.5%)	19	(15.3%)	57	(14.3%)	37	(11.1%)	29	(14.7%)	
**Current smoking**											
No	371	(99.2%)	122	(98.4%)	398	(99.5%)	330	(99.1%)	193	(98.0%)	0.428
Yes	3	(0.8%)	2	(1.6%)	2	(0.5%)	3	(0.9%)	4	(2.0%)	
**History of major diseases**^**b**^											
No	319	(85.3%)	104	(83.9%)	334	(83.5%)	287	(86.2%)	175	(88.8%)	0.491
Yes	55	(14.7%)	20	(16.1%)	66	(16.5%)	46	(13.8%)	22	(11.2%)	
**Depressive symptoms (GDS5)**											
Normal (< 2 points)	324	(86.6%)	104	(83.9%)	358	(89.5%)	284	(85.3%)	174	(88.3%)	0.340
Have depressive symptoms (≥2 points)	50	(13.4%)	20	(16.1%)	42	(10.5%)	49	(14.7%)	23	(11.7%)	
**Housework**											
Mainly do	365	(97.6%)	121	(97.6%)	252	(63.0%)	287	(86.2%)	149	(75.6%)	< 0.001
Not mainly do	9	(2.4%)	3	(2.4%)	148	(37.0%)	46	(13.8%)	48	(24.4%)	
**Social participation**											
Yes	327	(87.4%)	91	(73.4%)	342	(85.5%)	290	(87.1%)	172	(87.3%)	0.002
No	47	(12.6%)	33	(26.6%)	58	(14.5%)	43	(12.9%)	25	(12.7%)	
**Relationship with neighbors**											
Frequent	359	(96.0%)	112	(90.3%)	393	(98.3%)	328	(98.5%)	194	(98.5%)	< 0.001
Not frequent	15	(4.0%)	12	(9.7%)	7	(1.8%)	5	(1.5%)	3	(1.5%)	

^a^ Chi-square test

^b^ History of major diseases was defined as having any one of the following diseases: stroke, myocardial infarction/angina, diabetes, Parkinson’s disease, femoral neck fracture, and cancer

The original article [1] has been updated.
